# Recent Advances in Local Anesthesia: A Review of Literature

**DOI:** 10.7759/cureus.36291

**Published:** 2023-03-17

**Authors:** Bhumika J Patel, Pratik Surana, Keyur J Patel

**Affiliations:** 1 Department of Oral Medicine and Radiology, Howard University College of Dentistry, Washington, DC, USA; 2 Department of Pedodontics and Preventive Dentistry, Maitri College of Dentistry and Research Center, Durg, IND; 3 Department of Internal Medicine, Government Medical College & Hospital, Surat, IND

**Keywords:** pain management, local anaesthesia, recent trends, local anesthesia, pain

## Abstract

Even if local anesthetic is still the mainstay of pain management in dentistry, research will keep striving for novel and effective pain management techniques. The majority of research efforts are focused on improving anesthetic medications, delivery systems, and related methods. There are more recent technologies available that can assist the dentist in providing better pain relief with fewer unpleasant injections and fewer negative adverse effects. The purpose of the current review of the literature is to compile evidence that will convince dentists to employ modern local anesthetics, as well as other methods and techniques to reduce patient discomfort while administering anesthesia.

## Introduction and background

Without the use of local anesthetic, contemporary dentistry would be impossible. For the purpose of achieving local anesthetic, the dentist has access to a variety of tools and techniques. It is paradoxical, nevertheless, that while local anesthetic makes it possible for dental procedures to be done without feeling any pain, it also makes patients feel the most discomfort and fear [1].

In today's dentistry practice, pain management that is both safe and efficient is crucial. For the majority of clinical settings, our existing armamentaria for administering local anesthetic solutions to the maxilla and mandible are sufficient. For invasive dental procedures including cavity preparation, tooth preparation, scaling and root planing, surgical procedures, or essential pulp therapy, local anesthetics are employed. Local anesthetics are utilized for almost all dental procedures with the exception of examination, oral prophylaxis, and fluoride application, depending on the patient's tolerance for pain or level of fear [2].

Thus, numerous techniques have been advocated to diminish the pain during the administration of local anesthetic agents, and these include the application of different anesthetic gels [3], distraction techniques [4], warming the anesthetic agents [5], reducing the speed of injection [6], and buffering local anesthetic agents [7]. The current review of the literature's findings aims to compile solid data for dentists about the use of modern local anesthetics, alternative techniques, and tactics to lessen the pain when administering anesthesia, thereby enhancing patient comfort.

## Review

Recent advances in local anesthetic drugs

Articaine and centbucridine are two relatively recent medications that have been shown to be as effective as or perhaps more effective than lignocaine [8].

Mechanism of Action of Articaine

Articaine is a local anesthetic that is a member of the amide family. It has an ester group that is processed by tissues' esterases and a thiophene ring in place of a benzene ring. Articaine has an exponential half-life and is eliminated over an extended period of time. Unidentified plasma esterases are mostly responsible for metabolism in the liver and plasma [8].

Articaine Versus Lignocaine

Articaine has a faster onset of action and longer duration of action. Its success rate is greater. Articaine has more strong effects (1.5 times more potent) and has a lower level of systemic intoxication [8].

Adverse Effects of Articaine

Similar to prilocaine, articaine has the potential to produce neuropathies and methemoglobinemia. Articaine and prilocaine have increased paresthesia incidence, mainly with the lingual nerve, indicating that they have a more neurotoxic effect than lidocaine. It has been observed that taking articaine, particularly for infraorbital nerve block, can cause eye problems. The enhanced drug diffusion across tissues, including bone, may be the cause of this [8].

Centbucridine

Centbucridine is a local anesthetic molecule that was created in 1983 at Lucknow, India's Centre for Drug Research. It functions as a local anesthetic and is a quinolone derivative. It naturally contains antihistaminic and vasoconstricting effects. Centbucridine, which has an anesthetic power 4-5 times larger than that of 2% lignocaine, can be used successfully for infiltration, nerve blocks, and spinal anesthesia at a concentration of 0.5% [9].

Although clinicians have strangely failed to capitalize on its advantages and also validate its use in the management of pain during dental procedures, this unique chemical has been extensively used in ophthalmology and other medical disciplines [8]. Centbucridine, according to Gune and Katre [9], is comparable to lignocaine and can be used as a substitute in cases of hypersensitivity in patients aged 12-14, as well as in cases of cardiac and thyroid diseases where these vasoconstrictors are prohibited.

Alternative dental anesthesia

These techniques do not substitute conventional dental anesthesia. These techniques are used as an adjunct to conventional anesthesia to reduce pain during the administration of local anesthetics.

Electric Dental Anesthesia

It is a frequently employed non-pharmacological approach for treating both acute and ongoing pain. Transcutaneous electrical nerve stimulation (TENS) uses an electrical current generated by a machine to stimulate nerves, mostly for remedial purposes. Because the equipment does not include any syringes, it instills positive behavior in kids and lessens their apprehension. Hence, pediatric patients can benefit from this method. It can also be equally helpful for adult patients to produce analgesia during various conditions such as placing rubber dams, preparing cavities, capping pulp, performing endodontic procedures, preparing prosthetic teeth, performing oral prophylaxis, and extractions, and also to lessen pain during local anesthetic injection [10].

Laser Analgesia

Low-level laser therapy (LLLT) is used in a non-thermogenic, noninvasive procedure to biomodulate the tooth pulp. Similar to infiltrative local anesthesia, LLLT does not induce profound anesthesia or a total loss of sensation. The sodium-potassium (Na-K) pump is temporarily disrupted by the principle's modification of neuronal cell activity, which prevents impulse transmission and produces the analgesic effect [11]. Children and teens experience less anxiety as a result of accepting laser dental treatment [12]. The effectiveness of the neodymium-doped yttrium aluminum garnet (Nd:YAG) laser in inducing pulpal analgesia was confirmed by Chan et al. [13] to be comparable to that of 5% eutectic mixture of lidocaine 2.5% and prilocaine 2.5% (EMLA) anesthetic cream. Chan et al. also proposed that laser therapy may be a novel, noninvasive treatment option for children who are needle-phobic.

Virtual Anesthesia

The most often used behavioral strategies for reducing dental anxiety are distraction tactics. Virtual reality (VR) equipment is currently a more entertaining type of diversion. Despite these drawbacks, numerous researchers have claimed that it lessens discomfort and enhances patient satisfaction during treatment [14]. A decrease in pain and anxiety during pediatric dental treatments was reported in clinical investigations on VR [15]. These findings suggest that VR can be utilized as a complementary technique for non-pharmacological analgesia. This is known as "virtual anesthesia" because of the analgesic potential of VR. According to Atzori et al. [16] and Nunna et al. [17], VR is an effective method for assisting kids in dealing with dental fillings and extractions in a way that is less stressful and more enjoyable than its alternative.

Cryoanesthesia

This procedure involves cooling a constricted body area with ice or refrigerant sprays to prevent nerves from transmitting pain signals. Hence, the topical administration of cold would stimulate pain-inhibitory pain pathways and excite myelinated A-fibers. By reducing the threshold of tissue nociceptors and pain-carrying conduction nerve signals, cooling leads to neuropraxia [18]. According to Hindocha et al. [19], 5% lidocaine gel during needle insertion has the same effect as applying ice to the oral mucosa as a topical anesthetic prior to injection. After application, the topical anesthetic's effects persist for a few minutes [19]. Bose et al. [20] claimed that precooling the soft tissue area before routine dental operations decreases the pain perception for infiltrations and blocks anesthesia in youngsters. It is a simple, dependable, and economical technique. According to a comprehensive review by Tirupathi and Rajasekhar [21], precooling with ice before administering local anesthetic lowers pain more effectively than refrigerant spray [21].

Recent advances in local anesthesia delivery devices

The gate control hypothesis of pain management suggests that pain can be minimized by simultaneous activation of nerve fibers using vibration, and some of the more recent local anesthetic delivery devices intended to lessen needle phobia make use of this notion [22]. However, Inui et al. have shown that tactile-induced pain inhibition occurs without any input from the spinal level, including descending inhibitory actions on spinal neurons, and can result in pain reduction produced by non-noxious touch or vibration [23].

VibraJect

Recently, a vibrating dental local anesthetic attachment (VibraJect) was unveiled. It sends a strong enough high-frequency vibration to the needle for the patient to sense [23]. On the basis of the gate-control theory, interference stimulation, such as vibration, can reduce pain, according to Kakigi and Watanabe [24]. Hutchins et al. claimed that the vibration may be useful in lessening injection pain [25]. VibraJect was suggested by Blair [26] as a painless injection method.

DentalVibe

A recently developed device called DentalVibe vibrates during the administration of dental injections to stimulate the mechanoreceptors and lessen pain. The tool delivers percussive micro-oscillations to the injection administration site through a U-shaped vibrating tip that is handheld and cordless [27]. Ungor et al. [28] examined how DentalVibe affected adult patients' perceptions of pain and anxiety during local anesthetic injections. They discovered that DentalVibe lessened discomfort without elevating anxiety during local anesthetic injections.

Accupal

It is a cordless gadget that conditions the oral mucosa by using vibration and pressure together. The inventor of this tool is Michael Zweifler. Pressure is applied by Accupal, and it also vibrates the injection site 360 degrees around the point where the needle infiltrated and closed the "pain gate." The unit vibrates after being positioned at the injection site, and a light pressure is applied. The battery-powered motor is connected to the needle, which is positioned in a hole with a disposable tip head [29].

Buzzy System

The device is designed like a bee and has two components: detachable ice wings and body vibration. It functions according to the descending inhibitory controls and the gate control theory. More exactly, the vibration created by the device will obstruct the afferent pain-receptive fibers (A-delta and C fibers), which will reduce discomfort [30]. On the other hand, when administered close to the nociception location, continuous cold administration stimulates the C nociceptive fibers and suppresses the A-delta signals [31]. According to Suohu et al. [32], the Buzzy® System, which externally administers cold and vibration adjacent to the site of local anesthesia administration, can reduce children's pain and anxiety during the delivery of local anesthesia next to the tooth, which are indicated for invasive dental procedure.

Recent advances in local anesthesia delivery technique

Single Tooth Anesthesia (STA)

This technique uses a 30-G needle that is extra-short and inserted into the gingival sulcus parallel to the tooth's long axis. With single- and multi-rooted teeth, the number of locations for providing anesthesia is one point (distal) versus two (distal and mesial)/three points, respectively. To induce sufficient anesthetic, the needle must be inserted into the tissue up until it reaches the periodontal ligament (PDL). Due to its pen-like shape, Single Tooth Anesthesia has the advantages of not causing anticipatory worry, not hurting, having no effects on the lips, tongue, or cheeks, and not damaging the permanent teeth's crown [33]. STA is an efficient substitute for conventional treatments, according to Garret-Bernardin et al. [34], because it causes children less severe pain and distress.

Intranasal Tetracaine/Oxymetazoline Spray

Tetracaine is a water-soluble, long-lasting ester local anesthetic that, when given topically, has 5-8 times the potency of cocaine [1]. It is metabolized in plasma by plasma pseudocholinesterase, and a concentration of 0.15% and 2% are utilized as injection and topical application, respectively [35]. Before surgical and exploratory procedures, it is utilized as a nasopharyngeal and nasal anesthetic drug [36,37]. Hersh et al. [38] conducted a 150-person, double-masked, randomized, placebo-controlled research to assess the effectiveness and tolerability of 3% tetracaine/0.05% oxymetazoline (K305) intranasal spray for maxillary dental anesthetic. A third spray was also given if necessary after the first two 0.2 mL were sprayed into the ipsilateral nostril at a four-minute interval. With a 95% confidence interval, pulpal anesthesia had an overall success rate of 88% while performing restorative procedures on maxillary incisors, canines, and premolars.

Periodontal Ligament Injection

Intraligamentary injection (ILI), also known as the periodontal ligament injection, has been shown to be quite effective when only one tooth in the mandible needs to be sedated [39]. Only localized soft tissue anesthesia develops after the PDL injection administers pulpal anesthesia for the tooth. In contrast to a traditional inferior alveolar nerve block, when the jaw is injected, there is no extraoral or lingual numbness. Constraints include the likelihood of a local anesthetic solution to leak into the patient's mouth and the inability to properly determine the needle implantation position (inside or at the PDL entry). High pressure is required to inject the local anesthetic into the thick oral tissues at the PDL injection site using a standard syringe [40,41].

QuickSleeper

To lessen the pain of the anesthetic effect's injection, anesthesia is administered using this approach at a steady velocity and pressure [42]. It consists of a handpiece and a control box; by depressing the pedal, signals are transmitted through Bluetooth to the main control box. The handpiece drills and injects an anesthetic solution into the cancellous bone or intra-bony area after the circuit is complete to produce the greatest anesthetic effectiveness [43]. According to Smaïl-Faugeron et al. [44], the QuickSleeper® technology makes it easier for dental professionals to treat young patients and adolescents because it causes less discomfort and anxiety.

## Conclusions

Local analgesia is a secure and reliable pain management technique. One of the foundational tenets of contemporary dentistry practice is its application. Traditional methods for administering local anesthetics no longer seem to be as effective as modern approaches. These more modern methods are being promoted for their benefits and have a broad range of potential applications in dentistry. Modern techniques for providing local anesthetics efficiently and painlessly make the process more enjoyable for the dentist and the patient, which has a good impact on establishing a strong patient-dentist bond.
